# Supplementary material for: Photothermal Regulated Nanozyme of CuFeS2 Nanoparticles for Efficiently Promoting Wound Healing Infected by Multidrug Resistant Bacteria
Source: Nanomaterials (Basel). 2022 Jul 19;12(14):2469. doi: 10.3390/nano12142469 (PMC9315979; doi:10.3390/nano12142469)
Supplement: Supplementary file 1 [file nanomaterials-12-02469-s001.zip › nanomaterials-1789375-supplementary.pdf]

nanomaterials

Electronic Supplementary Information

Photothermal Regulated Nanozyme of $CuFeS_2$ Nanoparticles for Efficiently Promoting Wound Healing Infected by Multidrug Resistant Bacteria

Zezhong Liu †, Zengxu Liu †, Zhen Zhao, Danxia Li, Pengfei Zhang, Yanfang Zhang, Xiangyong Liu, Xiaoteng Ding * and Yuanhong Xu

Institute of Biomedical Engineering, College of Life Sciences, Qingdao University, 308 Ningxia Road, Qingdao 266071, China; lzz56066@163.com (Z.L.); zengxu1996l@163.com (Z.L.); zhaozhenciac@163.com (Z.Z.); ldxjy2018@163.com (D.L.); pfzhang0106@163.com (P.Z.); zyf183162@163.com (Y.Z.); z337047@163.com (X.L.); yhxu@qdu.edu.cn (Y.X.)

* Correspondence: xtding@qdu.edu.cn

† These authors contributed equally to this work.

1. Materials and Apparatus

1.1. Materials.

Ferrous sulfate ($FeSO_4{\cdot}7H_2O$), cupric chloride ($CuCl_2{\cdot}2H_2O$), and sodium sulfide (Na_2S) were bought from Sinopharm Chemical Reagent Co. (Shanghai, China); 5-tert-butoxycarbonyl 5-methyl-1-pyrroline N- oxide (BMPO), 3- (4,5-dimethyl-2-thiazolyl)-2,5-diphenyltetrazolium bromide (MTT), glucose, H_2O_2 solution (30 wt % aqueous), and dimethyl pyridine N-oxide (DMPO) were purchased from Sigma-Aldrich. Inc(Shanghai, China); 3,3′,5,5′-tetramethylbenzidine (TMB), o-phenylenediamine (OPD), and 4- nitrobenzoic acid (DTNB) were purchased from the Shanghai Yuanye Bio-Technology Co. Ltd. (Shanghai, China), 2, 2'-azino-bis(3-ethylbenzothiazoline-6-sulfonic aci (ABTS) was bought from Sigma-Aidrich Co. Ltd. (Shanghai, China), riboflavin (MW 376.36, 98%) was purchased from Alfa Aesar Co. propidium iodide (PI), and Luria-Bertani (LB) medium and STYO 9 were purchased from Thermo Fisher Scientific Inc (Beijing, China). Murine L929 cells were bought from the Affiliated Hospital of Qingdao University (Qingdao, China). Methicillin-resistant Staphylococcus aureus (MRSA), extended-spectrum β- lactamase (ESBL)-producing Escherichia coli (ESBL-E. coli), and Psedomonas aeruginosa (PA) were bought from the Experimental Teaching Centre for Biology in Qingdao University (Qingdao, China).

1.2. Apparatus

Scanning electron microscope (SEM) image was taken by an S-4800 (Hitachi, Japan). The UV absorption spectra were recorded using a Mapada UV-6300 spectrophotometer (Shanghai, China). The high-resolution TEM (HRTEM) and transmission electron microscopy (TEM) were were recorded on a JEOL JEM-2100 microscope instrument at an acceleration voltage of 200 kV. X-ray photoelectron spectroscopy (XPS) measurements were conducted with a PHI5000 Versaprobe-II spectrometer. X-ray diffraction (XRD) characterizations were implemented on a

Bruker D8 advance diffractometer, with copper Kα (λ =0.154056 nm) radiation source. The fluorescence images were taken via a Leica TCS SP8 CARS fluorescence confocal microscope. The photoluminescence (PL) spectra were carried out on an FS5 spectrophotometer

2. Additional experimental section

2.1. Photothermal properties of $CuFeS_2$.

Firstly, $CuFeS_2$ NPs sample suspensions with different concentrations (62.5, 125, 250, 500, and 1000 μg/mL) were prepared, and PBS (pH 7.4, 0.1 M) was used as blank control. The power of the near-infrared 808 nm laser was set as 1.0 W/cm^2, and the $CuFeS_2$ NPs samples of different concentrations were irradiated for 360 s, with PBS as the blank group. Lighting was timed, and the temperature of the sample was recorded every 30 s with an infrared thermal imager. $CuFeS_2$ NPs (250 μg/mL) was tested at room temperature at 25℃ for 5 consecutive cycles of 10 min for each cycle (5 min for light and 5 min for natural cooling).

According to previous research reports, the photothermal conversion efficiency (η) of $CuFeS_2$ NPs was calculated as follows [1]: the prepared $CuFeS_2$ NPs solution, with a concentration of 250 μg/mL, was irradiated by an 808 nm near-infrared laser (1 W/cm^2), and the laser was turned off and cooled to room temperature when the temperature rose to a steady state. The temperature was recorded in the process of heating and cooling by infrared thermal imager. Calculate η value according to the calculation formula in Eq. 1:

$$\eta = \frac{hS(T_{\max} - T_{surr}) - Q_0}{I(1-10^{-A808})} \quad \text{(S1)}$$

In Equation 1, H is the heat transfer coefficient, S is the surface area of the sample container, T_{max} is the highest steady-state temperature, Q_0 is the reference energy input by the solvent without $CuFeS_2$ and the sample container, T_{surr} is room temperature, A808 is the absorbance value of the $CuFeS_2$ sample at 808 nm, and I is the power of the light source set by the near- infrared exciter. In Eq. 2, τs is the characteristic thermal time constant, Md is the mass of $CuFeS_2$ sample, and Cd represents its heat capacity, which is approximately the heat capacity of water. The value of *hs* is calculated from Eq.2:

$$\tau_s = \frac{m_d c_d}{hs} \quad \text{(S2)}$$

In Eq. 3, τs is the characteristic thermal time constant, Md is the mass of $CuFeS_2$ sample, and Cd represents its heat capacity, which is approximately the heat capacity of water.

$$Q_0 = hs(T_{\max} - T_{surr}) \quad \text{(S3)}$$

2.2. Glutathione depletion.

The $CuFeS_2$ NPs sample solution was co-incubated with glutathione (GSH, 0.3 mM) in 1 mL phosphate buffer (0.1 M, pH 7.4) at 37℃. At different incubation time points (0 h, 1 h, 3 h, and 6 h); 10 μL DTNB ethanol solution (1.0 mg/mL) was added into the mixed system, and the UV–VIS absorption spectra in the wavelength range of 300~600 nm were measured after shock mixing, and the characteristic peak was at 412 nm.

3. Additional Figures and Tables

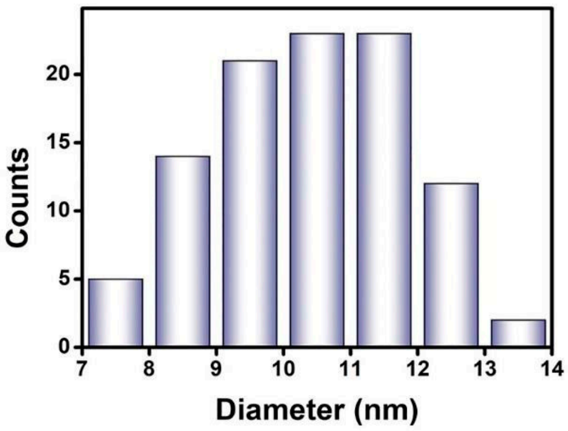

Figure S1. Diameter distribution of $CuFeS_2$ NPs. The average diameter of 10.38 ± 1.39 nm, as calculated from counting 100 particles.

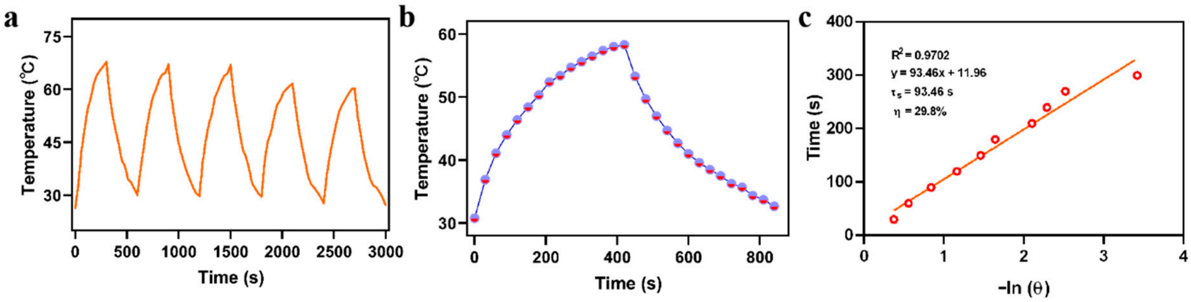

Figure S2. (a) Temperature cycling test of $CuFeS_2$ NPs. (b) The curve of single heating and cooling. (c) Linear relationship between -ln (θ) and time in cooling period.

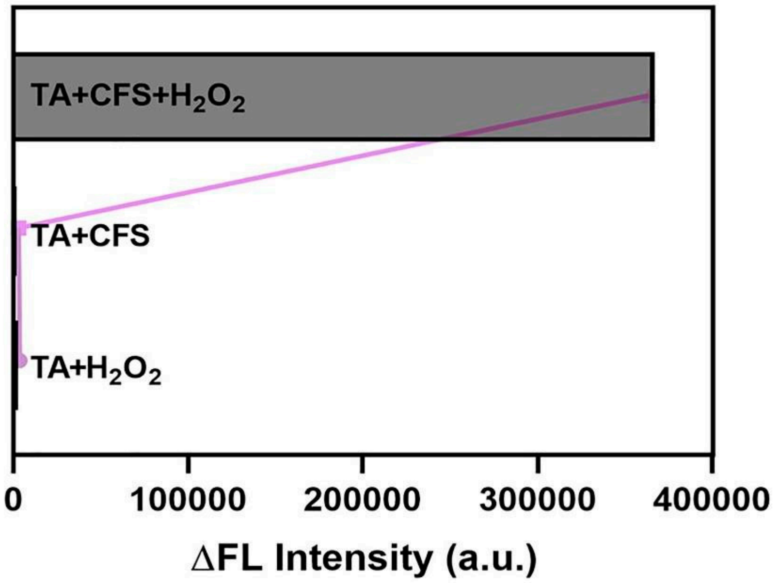

Figure S3. ΔPL intensity showed the catalytic activity of $CuFeS_2$.

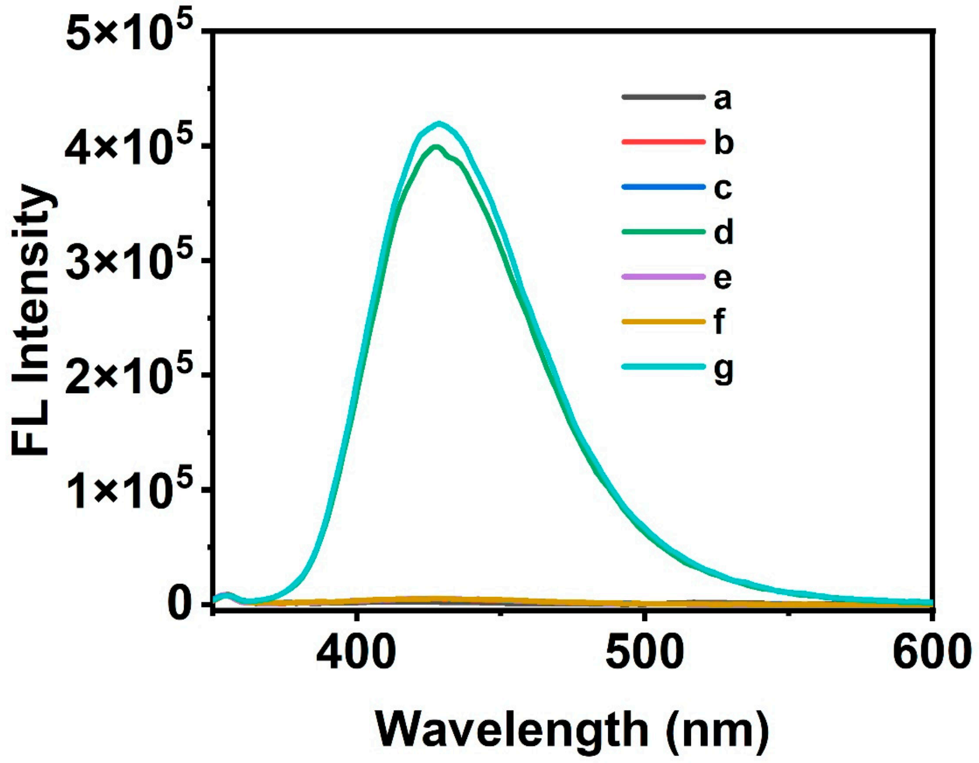

Figure S4. After irradiation by NIR, the fluorescence intensity was detected by TA method (a: TA, b: TA+H_2O_2, c: TA+$CuFeS_2$, d: TA+H_2O_2+$CuFeS_2$, e: TA+H_2O_2+NIR, f: TA+Cu FeS_2+NIR, g: TA+H_2O_2+$CuFeS_2$+NIR).

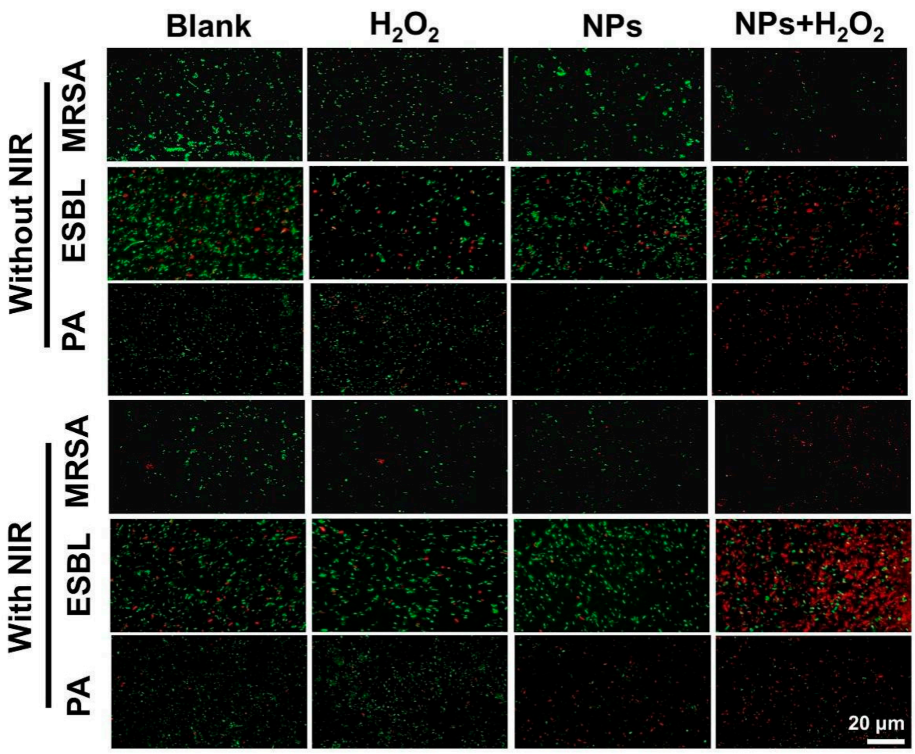

Figure S5. Fluorescence images of live/dead bacteria under different processing conditions. Bar scale: 20 μm.

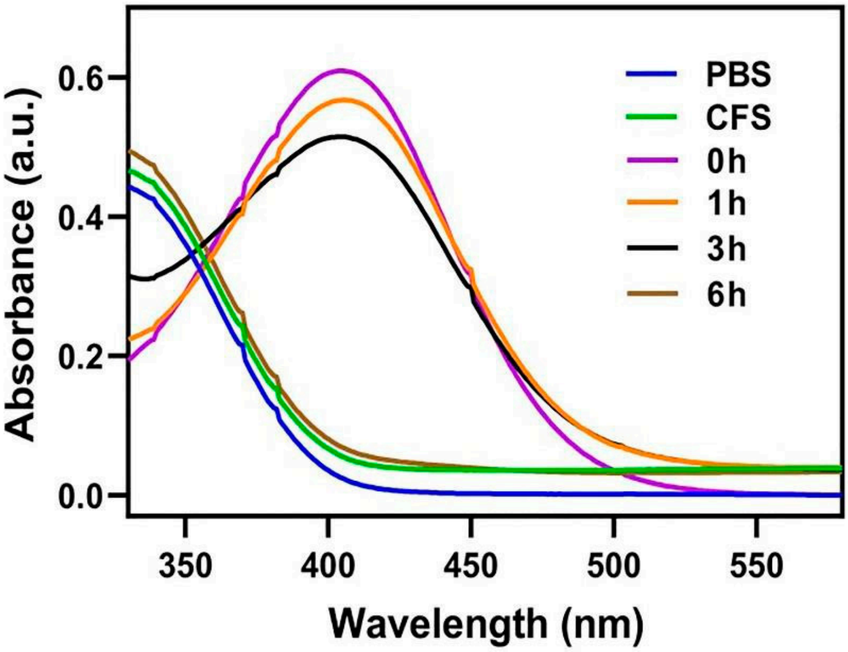

Figure S6. Evaluation of GSH consumption capacity of $CuFeS_2$ NPs at different times.

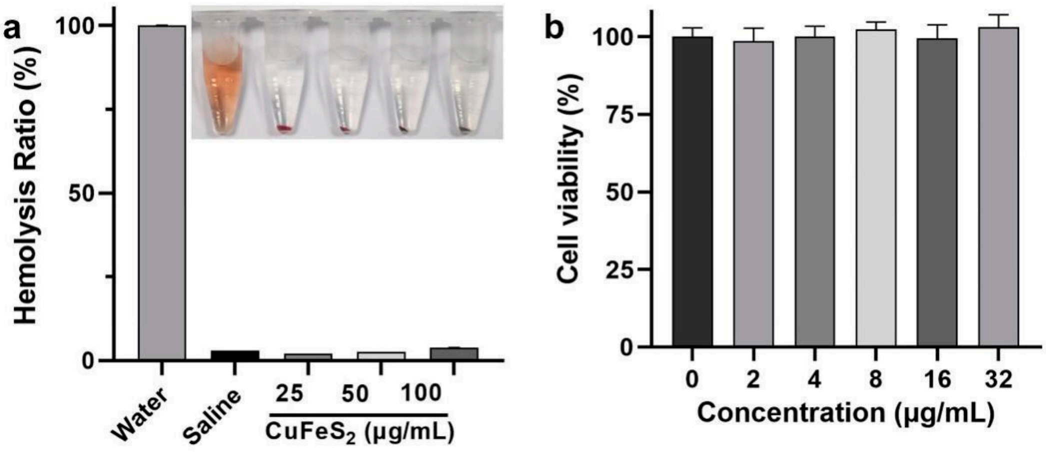

Figure S7. (a) Hemolysis ratio of fresh rat red blood cells (RBCs) incubated with $CuFeS_2$ NPs for 3 h (inset: RBCs incubated with water, saline, and different concentrations (25, 50, and 100 μg/mL) of $CuFeS_2$ for 3 h). (b) The cell viability of L929 cells cultured in different concentrations of $CuFeS_2$ NPs for 24 hours.

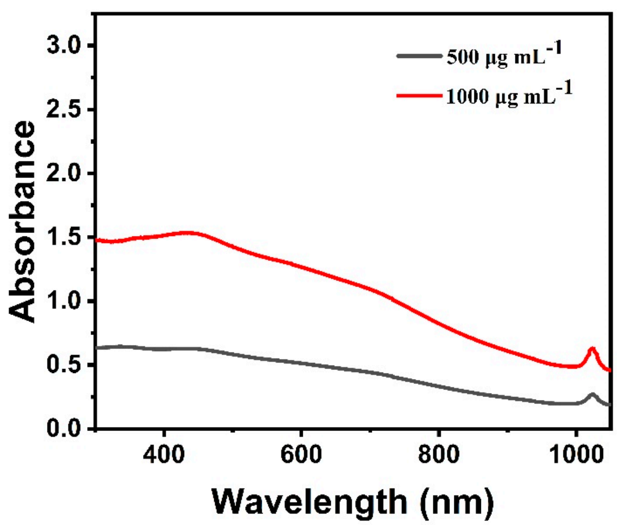

Figure S8. Visible−NIR absorption of $CufeS_2$.

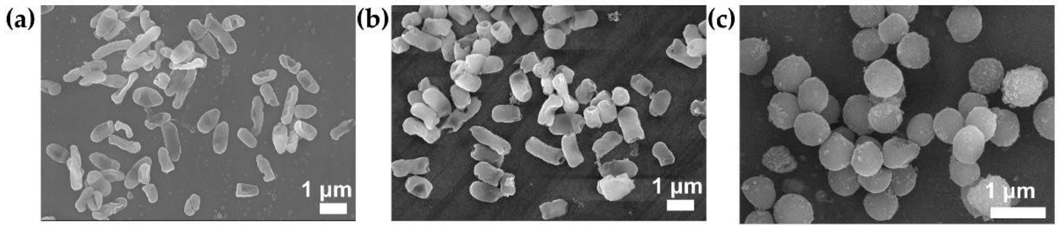

Figure S9. The SEM images of (a) PA, (b) ESBL, and (c) MRSA treated with $CuFeS_2$+NIR+H_2O_2.

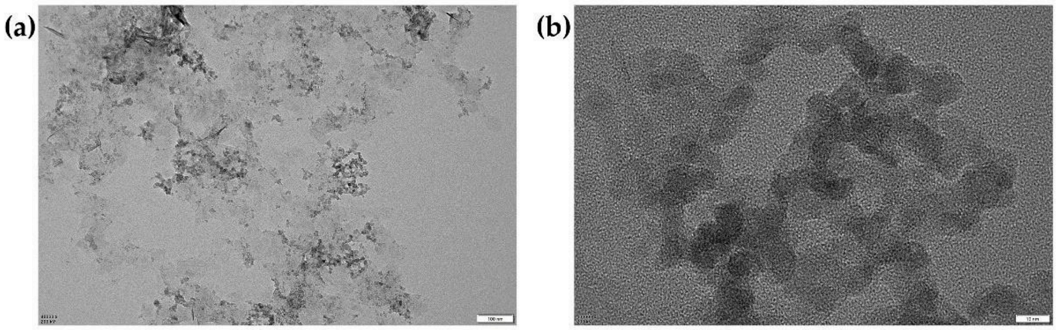

Figure S10. Different-sized (a) TEM and (b) HRTEM images.

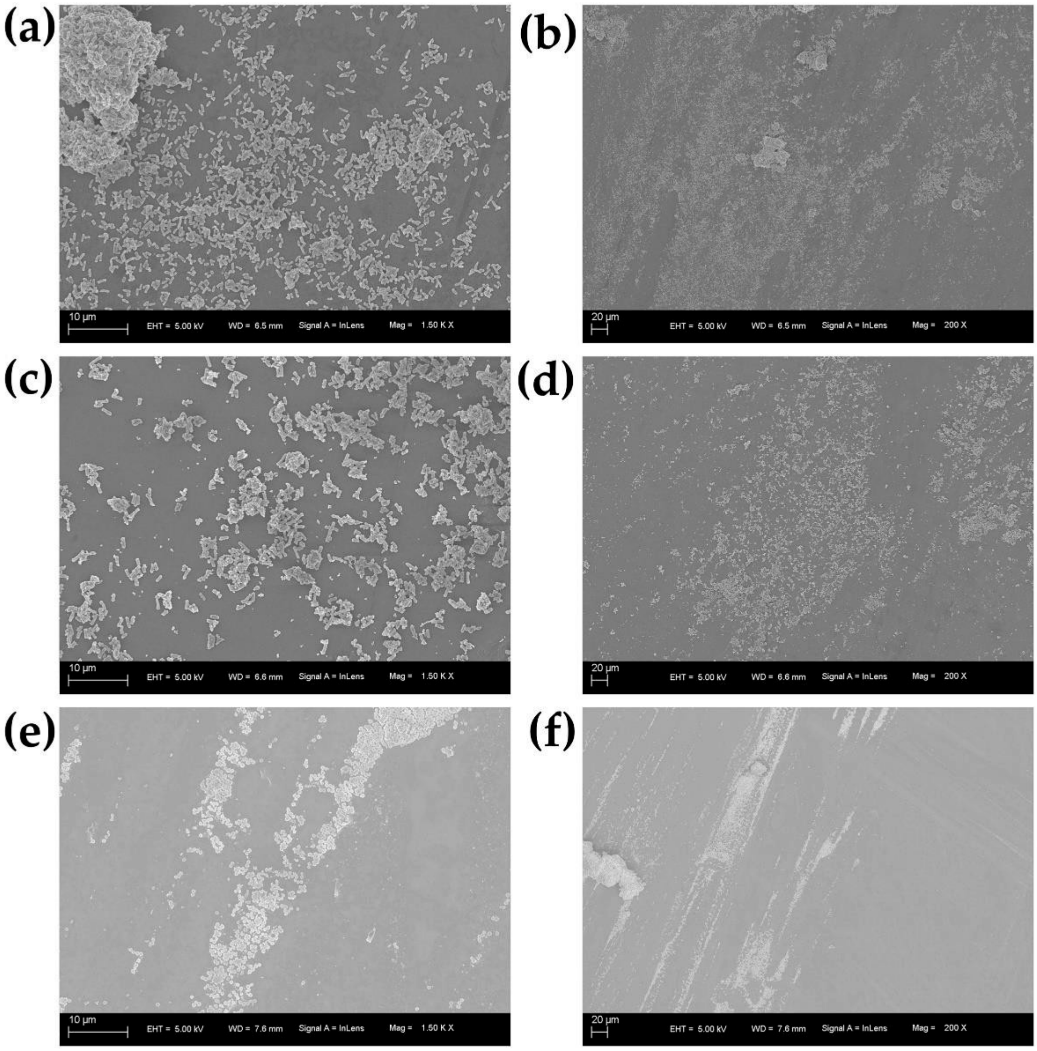

Figure S11. The SEM images of (a) and (b) PA, (c) and (d) ESBL, and (e) and (f) MRSA treated with $CuFeS_2$ +H_2O_2.

Table S1. The Michaelis–Menten constants (K_m) and maximum initial reaction rates (V_{max}) of the $CuFeS_2$, HRP, and other classical peroxidase-mimic activity.

Catalyst	Km (mM)		Vmax (10^{-8} M s^{-1})		References
	H2O2	TMB	H2O2	TMB	
HRP	3.7	0.434	8.71	10	[2]
Fe3O4	154	0.098	9.78	3.44	[2]
FeS2	0.30	0.17	5.62	3.93	[3]
CuFeS2	0.000091	0.014	2.05	24.6	This work

Additional Reference

[1] Zhang, Y.; Li, D.; Tan, J.; Chang, Z.; Liu, X.; Ma, W.; Xu, Y. Near-infrared regulated nanozymatic/photothermal/photodynamic triple-therapy for combating multidrug-resistant bacterial infections via oxygen-vacancy molybdenum trioxide nanodots. *Small* **2021**, *17*, 2005739.

[2] Gao, L.; Zhuang, J.; Nie, L.; Zhang, J.; Zhang, Y.; Gu, N.; Wang, T.; Feng, J.; Yang, D.; Perrett, S.; Yan, X. Intrinsic peroxidase-like activity of ferromagnetic nanoparticles. *Nature Nanotechnology* **2007**, *2*, 577-583.

[3] Song, C.; Ding, W.; Zhao, W.; Liu, H.; Wang, J.; Yao, Y.; Yao, C. High peroxidase-like activity realized by facile synthesis of FeS2 nanoparticles for sensitive colorimetric detection of H2O2 and glutathione. *Biosensors and Bioelectronics* **2020**, *151*, 111983.
